# Covid-19-Associated Mucormycosis: Histopathology of the Deadly Fungal Infection

**DOI:** 10.1055/s-0043-1776729

**Published:** 2024-01-24

**Authors:** Nidhi Anand, Pallavi Srivastava, Ashish Chandra Agrawal, Nikhil Gupta, Anupam Das, Nuzhat Husain

**Affiliations:** 1 Department of Pathology, Dr. Ram Manohar Lohia Institute of Medical Sciences, Lucknow, Uttar Pradesh, India; 2 ENT Department, Dr. Ram Manohar Lohia Institute of Medical Sciences, Lucknow, Uttar Pradesh, India; 3 Department of General Medicine, Dr. Ram Manohar Lohia Institute of Medical Sciences, Lucknow, Uttar Pradesh, India; 4 Department of Microbiology, Dr. Ram Manohar Lohia Institute of Medical Sciences, Lucknow, Uttar Pradesh, India

**Keywords:** COVID-19, histopathology, mucormycosis, rhino-orbital-cerebral

## Abstract

**Introduction** Many patients suffered from rhino-orbital-cerebral mucormycosis during the coronavirus disease 2019 (COVID-19) pandemic in India. Diabetes is a known risk factor of COVID-19 infection and mucormycosis.

**Objective** The present study was done to describe the clinical spectrum and histopathological findings of mucormycosis in COVID-19 patients and their outcomes.

**Methods** A cross-sectional study was done over a period of two and half months. The biopsy samples or scrapings from sinonasal or periorbital tissue of 38 patients were analyzed. Hematoxylin & Eosin (H&E stain) slides were evaluated along with Grocott-Gomori methenamine–silver and Periodic acid–Schiff stains to highlight the fungal elements.

**Results** The male to female ratio was 2.5:1, and the mean age of the subjects was 53 years old. A total of 68.4% ( *n* = 26/38) of the patients had diabetes as a comorbidity, 84.2% ( *n* = 32/38) had a history of steroid intake, and 55.3% ( *n* = 21/38) were given supplemental oxygen during their treatment. The common presentations were nasal blockage, discharge, eye pain, headache, and altered mentation. The sites of biopsy were: nasal cavity 76.3% ( *n* = 29/38), periorbital fat/orbit 21.1% ( *n* = 8/38), maxillary sinus 15.8% ( *n* = 6/38) and ethmoid sinus 13.2% ( *n =* 5/38). In 76.3% ( *n* = 29/38) cases, broad, irregular, nonseptate, and right-angle branching hyphae were seen on H&E-stained tissue sections.

**Conclusion** COVID-19 led to various complications in individuals affected by it. Mucormycosis was one such lethal complication. An early diagnosis and prompt treatment is crucial to control the progression of the disease and improve outcomes.

## Introduction

The Severe Acute Respiratory Syndrome Coronavirus 2 (SARS-CoV-2) has caused a global pandemic of Coronavirus disease 2019 (COVID-19). Its clinical presentation has been variable, ranging from dry cough with or without high-grade fever to various multisystem problems and secondary infections. A good number of patients who have recovered from the acute infection continue to suffer from multiple post-COVID complications. There has been an increased incidence of fungal infections in such patients, such as COVID-associated pulmonary aspergillosis (CAPA) and candidemia. 1 2 3 The sudden surge of mucormycosis in COVID-19 patients has led to widespread morbidity and mortality. 4

Mucormycosis is a serious disease caused by a group of moulds called mucoromycetes. It is a life-threatening angioinvasive opportunistic fungal infection that generally affects immunocompromised individuals with an impaired neutrophilic response. The vast presentation of mucormycosis involves a rhino-orbital-cerebral, pulmonary, cutaneous, gastrointestinal, and disseminated form of the disease. The common features associated with it are unilateral facial swelling, fever, headache, nasal or sinus congestion, black eschar in the nose or palate, ptosis, chemosis, ophthalmoplegia, and various neurological signs and symptoms if an intracranial extension is present. 5 6

Uncontrolled diabetes, acquired immunodeficiency syndrome, iatrogenic immunosuppression, post organ transplant and malignancy are the common risk factors for mucormycosis. 7 Critically ill patients admitted to intensive care units (ICUs) or who have a longer hospital stay are more likely to develop fungal coinfections. 8 The widespread use of steroids in the management of COVID-19 led to the development of opportunistic fungal infections.

Early diagnosis and treatment are crucial to control the rapid progression of the disease and to improve outcomes. Studies have shown that timely initiation of antifungal agents may reduce the requirement or extent of surgical resection, disfigurement, and increases the survival. 9 10 A diagnostic workup consists of the identification of risk factors, assessment of clinical manifestations, usage of imaging techniques, sampling based on histopathology, cultures, and advanced molecular techniques.

Histopathology is an important diagnostic tool as it distinguishes the presence of the fungus as a pathogen in the specimen from a culture contaminant and identifies the blood vessel invasion. 11 It also can identify coinfection with other moulds. Routine hematoxylin and eosin (H&E) stains highlight only the cell wall, or occasionally degenerate hyphae. To highlight the fungal wall, special stains like Grocott methenamine-silver (GMS) and Periodic acid-Schiff (PAS) are useful. Periodic acid-Schiff gives a better visualization of the surrounding tissue compared with GMS. 11 A rapid presumptive diagnosis of mucormycosis can be made on direct microscopy of potassium hydroxide (KOH) mounts. However, its limitation is that it does not identify the fungus to the genus or species level. The culture of specimens is helpful in identification of the genus and species and in antifungal susceptibility testing. There are possibilities of false positive results, especially when histopathology is not available. Therefore, histopathology is recommended along with wet mount, by a panel of experts of the European Confederation of Medical Mycology in cooperation with the Mycoses Study Group Education and Research Consortium (ECMM/MSG ERC). 12 With the aforementioned background, the present study was done to describe the clinical spectrum and histopathological findings of mucormycosis in patients with COVID-19 and their subsequent outcomes.

## Method

A cross sectional record-based study was done over a period of two and half months, from May to mid-July 2021, at a tertiary care teaching institute of north India. An approval of the institutional ethics committee (IEC No. 90/20) was obtained prior to the start of the study. An individual consent for the study was waived. A total of 38 patients of sinonasal invasive mucormycosis were considered for the study. The study participants were either COVID- 19 positive or had recovered from its infection and presented to the ENT department either as an outpatient or by a referral from another department. The inclusion criteria were: 1) Patients of all ages and gender, 2) cases of acute invasive fungal rhinosinusitis based on clinical evaluation, nasal endoscopy examination, computed tomography (CT) scan/ magnetic resonance imaging (MRI) scan findings. After a written informed consent, the sample was collected from a representative site within the nasal cavity (after keeping cotton pledgets soaked in 4% xylocaine for 10 minutes) under the vision of an endoscope and was used for making a diagnosis. Potassium hydroxide mount, fungal culture, gram stain, and bacteriological culture were performed in the microbiology department and histopathologic examination was performed in the pathology department. The clinical presentation, radiological findings, comorbidities, management details, and follow-up details of the patient were also recorded and analyzed.

For histopathological evaluation, the tissue samples received in the department of pathology were fixed in 10% formalin and paraffin-embedded tissue blocks were prepared. Sections of 3 to 4 µm were cut and stained with H&E stain. These slides were evaluated under the microscope using low and high-power resolution. Special stains like GMS and PAS were used to highlight the fungal elements.

The data was compiled in Microsoft Excel (Microsoft Corporation, Redmond, WA, USA) and analyzed using IBM SPSS Statistics for Windows version 20.0 (IBM Corp., Armonk, NY, USA). Descriptive statistics were obtained as mean and standard deviation (SD) for quantitative variables and as frequencies with percentages for qualitative data.

## Results

### Demographics

The study included 38 patients, out of which 27 were males and 11 females. The age ranged from 32 to 78 years with a mean age of 53 years. A total of 34 patients had recovered from COVID-19 infection and the remaining 4 were COVID-19 positive at the time of presentation and had been infected for > 14 days.

### Risk Factors

In the present study, 68.4% ( *n* = 26) patients had diabetes as a comorbidity. A total of 84.2% ( *n* = 32) of the patients had a history of steroid intake. There were no patients who had an organ transplant, malignant condition, or HIV infection. At the time of diagnosis of mucormycosis, 55.3% ( *n* = 21) of the patients had hypoxemia and required supplemental oxygen. ( Table 1 ) Three patients had received a single dose of the COVID-19 vaccine (Covishield).

**Table 1:** Clinicopathologic and histological features of cases included in the study

S.N.	Parameters	Total number of cases *n* = 38 (%)	Survivors ( *n* = 34) (%)	Non survivors ( *n* = 4)	*p-value*
**1.**	**Age (years old)**				
	< 40	7 (18.4)	06 (85.7)	1 (14.3)	0.7197
	➣ 40	31 (81.6)	28(90.3)	3 (9.7)	
**2.**	**Site of biopsy**				
	Nasal cavity	29 (76.3)	28	01	0.124
	Maxillary sinus	06 (15.8)	06	00	
	Ethmoid sinus	05 (13.2)	04	01	
	Orbit and periorbital fat	08 (21.1)	06	02	
**3.**	**History of diabetes**				
	Yes	26 (68.4)	24 (92.3)	02 (7.7)	0.4020
	No	12 (31.6)	10 (83.3)	02 (16.7)	
**4.**	**History of Steroid intake**				
	Yes	32 (84.2)	29(90.6)	03(9.4)	0.5932
	No	06 (15.8)	05(83.3)	01(16.7)	
**5.**	**Oxygen therapy**				
	Yes	21 (55.3)	17(81.0)	04 (19.0)	0.1133
	No	17 (44.7)	17(100)	00 (0)	
**6.**	**Direct microscopy by KOH mount**				
	Yes	35(92.1)	31(88.6)	04(11.4)	1.00
	No	03(7.9)	03(100)	00 (0)	
**7.**	**Diagnosis by culture**				
	Yes	26(68.4)	25 (96.2)	01(3.8)	0.048
	No	12(31.6)	09(75)	03(25)	
**8.**	**Histological diagnosis of fungus infestation**				
	Yes	29 (76.3)	28(96.6)	01(3.4)	0.0107
	No	09 (23.7)	06(66.7)	03(33.3)	
**9.**	**Presence of angioinvasion on histopathology**				
	Yes	06 (15.8)	03(50)	03(50)	0.0005*
	No	32 (84.2)	31(96.9)	01(3.1)	

Abbreviation: KOH, potassium hydroxide.

### Clinical Presentation

**Fig. 1:**
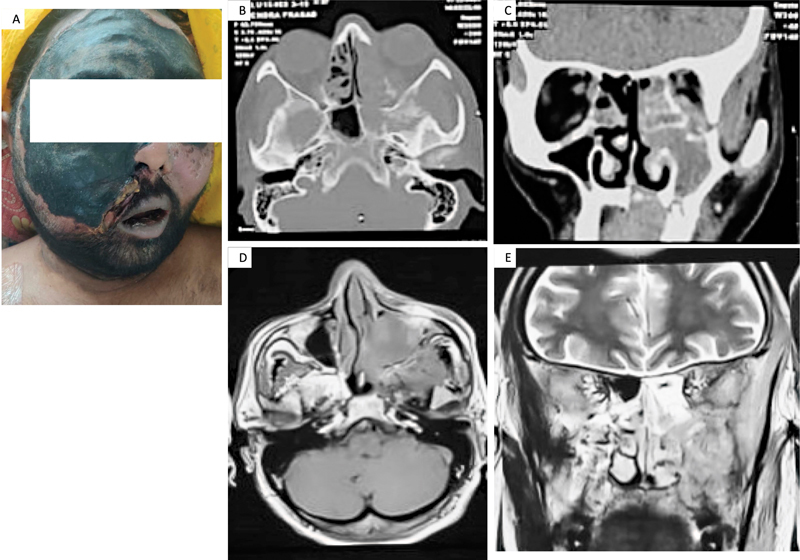
( **A** ) Clinical image of an affected patient with extensive facial swelling and blackish discoloration. ( **B, C** ): Computed tomography scan images showing heterogenous opacification of all the paranasal sinuses of the left side. There is destruction of the walls of the maxillary sinus with the involvement of the pterygopalatine, infratemporal fossa and orbit by the disease. ( **D, E** ): Contrast-enhanced magnetic resonance imaging images showing bilateral mucosal thickening of the paranasal sinuses (more on the left side). There is an enhancement on the administration of contrast.

In the present study, the most common clinical presentation of fungal infestation in post-COVID patients was related to the nasal cavity, in the form of nasal blockage, runny nose, reduced or absent sense of smell and external blackish discoloration ( Figure1A ). Clinical presentations related to eye involvement were eye pain, reduced or absent vision or altered color perception, double vision, watering from the eye, and redness around the eye. The patients with intracranial spread clinically presented with headaches and altered mentation. Patients with maxillary involvement presented with loosening of teeth and regurgitation of food/fluids through the palatal fistula. On examination of these patients, black crust/eschar was seen in the nasal cavity ± paranasal sinuses, purulent secretions, and pale nasal mucosa (in early presenting cases).

### Radiological Findings

Noncontrast CT scan of face showed mucosal thickening, opacification of paranasal sinuses, local destruction of the surrounding structures by the disease, including the maxillary sinus, the pterygopalatine, the infratemporal fossa, and the orbit. ( Figure 1B, C ) Contrast-enhanced MRI was performed in a few cases, revealing mucosal thickening of the paranasal sinuses and some showing contrast enhancement. ( Figure 1D, E )

### Histopathological Findings

**Fig. 2:**
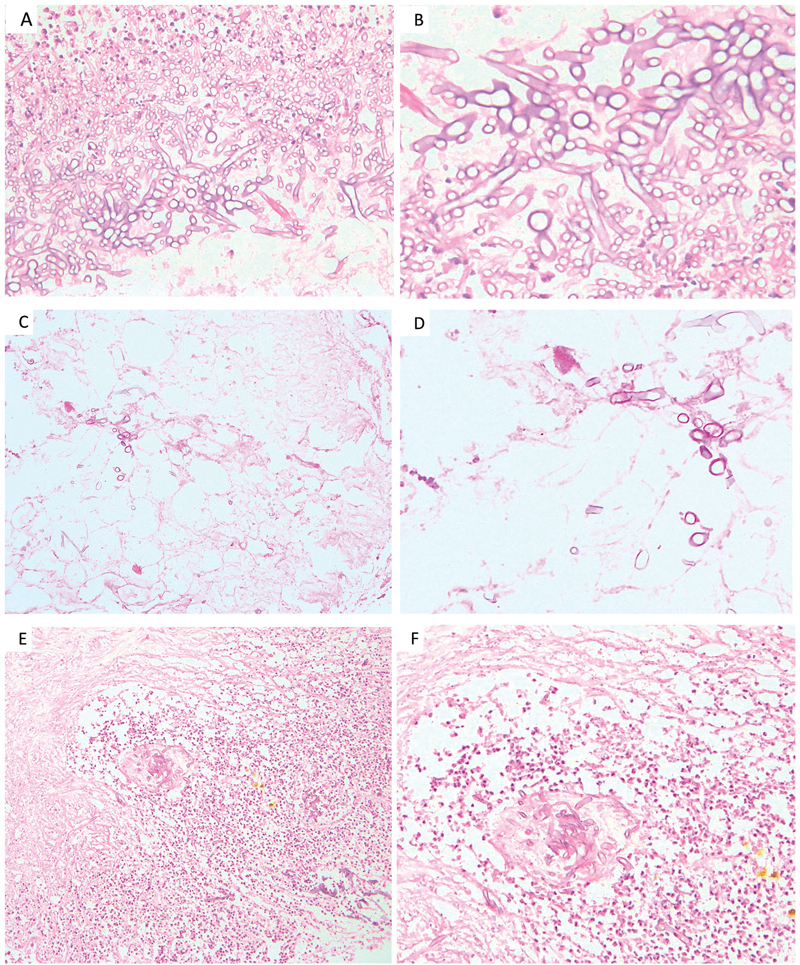
Photomicrograph ( **A, B** ) showing invasive broad aseptate hyphae characteristic of mucor in the background of inflammation. ( **C, D** ) showing fungal hyphae invading the periorbital fat. ( **E, F** ) shows angioinvasion by fungal elements [Hematoxylin-eosin; original magnification 100x (A, C, E); 200x (B, D, F)].

**Fig. 3:**
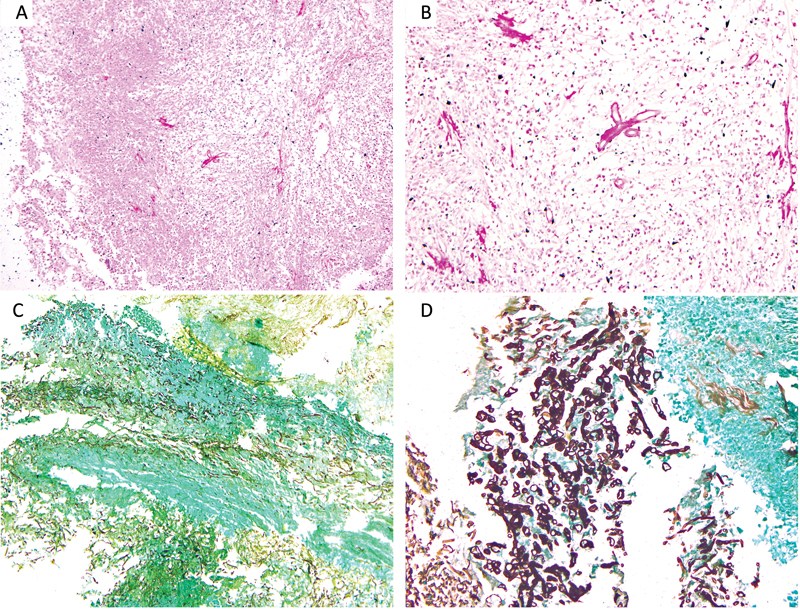
Photomicrograph ( **A-D** ) showing invasive broad aseptate hyphae characteristic of mucor in the background of the inflammation [PAS stain; original magnification 100x (A), 200x (B) GMS stain, 100x (C,) 200x (D)].

In the current study, the most common site of biopsy was from the nasal cavity (76.3%; *n* = 29/38), followed by periorbital fat and orbit (21.1%; *n* = 8/38), the maxillary sinus (15.8%; *n* = 6/38), and the ethmoid sinus (13.2%; *n =* 5/38) ( Table 1 ). In 76.3% ( *n* = 29/38) cases, broad, irregular, aseptate, and right-angle branching hyphae were seen on H&E-stained tissue sections. ( Figure 2A-D ). Angioinvasion was present in 15.8% ( *n* = 6/38; *p* < 0.05) cases ( Figure 2E, F ). A diagnosis of mucormycosis was made by clinical-radiological suspicion and the visualization of broad branched aseptate fungal hyphae on histopathology specimen by fungal stains. ( Figure 3A to 3D ).

In 23.7% ( *n* = 9/38) of the cases, we were unable to find any fungal elements on the tissue section even after multiple serial sectioning of the tissue. However, these were positive on fungal culture and responded to antifungal therapy. Out of these nine negative cases on histology, six cases were true negative as the parent tissue (sinonasal mucosa or periorbital adipocytes) was identified on histology along with chronic inflammatory cell infiltrate and necrosis. But in three cases, only fibrocollagenous tissue infiltrated by inflammatory cells was identified, so they were regarded as a false negative. These negative cases might be due to inappropriate biopsy size, or the biopsy was not taken from the representative site.

### Treatment and Outcomes

If the clinical features were strongly suspicious (black crusting/eschar formation/destruction of the palate or of the eye) or the histopathology was positive for mucormycosis or microbiological evaluation (KOH mount/culture) pointed towards fungal infestation, empirical antifungal treatment was started and surgical intervention in the form of endoscopic debridement was done at the earliest. If the situation warranted, an orbital exenteration/maxillectomy was performed. For antifungal treatment, liposomal amphotericin B at the dose of 3 to 5 mg/kg/day (up to 10 mg/kg, if there was intracranial involvement) was given parenterally. Most patients showed good recovery after 4 to 6 weeks of treatment. During the treatment, kidney function tests were monitored regularly. During the entire duration of the study, 10.5% ( *n* = 4/38) of the patients did not survive. The causes of death were intracranial complications (brain stem involvement, cerebral abscess, and cavernous sinus thrombosis), pulmonary involvement (pneumonia), septicemia, and fluid-electrolyte imbalance.

## Discussion

The burden of mucormycosis is showing a growing trend. 13 According to a study, the prevalence of mucormycosis in India is 140 per million individuals. 14 According to an article, its incidence was 0.27% amongst the hospitalized cases during the study period from September 2020 to December 2020. The case load of mucormycosis has increased by 2.1-fold from the previous years. 15 Around 14,872 cases of mucormycosis in COVID or postCOVID patients have been reported in India as of May 28, 2021. 4

Diabetes, as a comorbidity in mucormycosis, is found in ∼ 73.5% of cases in India. 16 However, in western countries, diabetes is associated with 17% of the cases of mucormycosis. 17 According to Bhansali et al., in 1,000 patients with diabetes, ∼ 1.6 present with mucormycosis. 18 In the present study, diabetes was associated with 68.4% ( *n* = 26/38) of the patients with mucormycosis. Clinically and radiologically, the predominant site of involvement and biopsy was the nasal cavity (76.3%; *n* = 29/38), followed by the orbit ( *n* = 8/38), and the paranasal sinuses. These findings are supported by few other reports in which the predominant risk factor was diabetes. 19 The mortality rate in the present study was 10.5% ( *n* = 4/38). In a study conducted by Roden et al., the mortality rate was 12.5% ( *n =* 4/32) and in those with cerebral involvement the mortality was between 40 and 50%. 19

The prognosis is improved in cases of sinonasal disease with an early surgical debridement and the reported mortality is < 10%. 20 In the present study, a lower mortality was observed, possibly due to an early diagnosis and a prompt surgical debridement along with antifungal treatment. In cases of mortality, the causes of death are intracranial complications, lung involvement, septicemia, and fluid-electrolyte imbalance. There are various reasons for the development of mucormycosis in COVID-19. In the present study, the most common comorbidity associated with COVID-19 was diabetes. Diabetes is a proinflammatory state which causes deficient control of SARS-CoV-2 replication and severe COVID-19 infection. 21 SARS-CoV-2 infection causes decreased insulin secretion because of the direct pathogenic effect on pancreatic islet cells. It also causes insulin resistance due to a transient hyperinflammatory state. 22 Eventually, a state of hyperglycemia is developed leading to the growth of invasive mucormycosis.

According to a study, 8% of COVID-19-positive or recovered patients were found to have secondary bacterial or fungal infections. 23 The possible reasons for this association could be the immunosuppression caused by COVID-19 infection, or the extensive use of steroids and broad-spectrum antibiotics for the management of COVID-19, leading to the development or aggravation of a pre-existing fungal disease.

In patients with COVID-19 on supplemental oxygen, corticosteroids have been extensively used. 24 Although a high dose is considered a risk factor for mucormycosis, few case reports have shown its occurrence even after a short course of steroids. 25 Corticosteroids have multiple effects on COVID-19 patients with associated mucormycosis. They can cause immunosuppression due to the impaired migration, phagocytosis, and phagolysosome formation in the macrophages. They also cause drug-induced hyperglycemia and worsening of glycemic control in patients with diabetes.

SARS-CoV-2 infection causes deficient innate immunity by immune dysregulation and a cytokine storm by infecting immune cells (CD3, CD4, and CD8 T cells). 26 On the other hand, diabetes mellitus affects adaptive immunity by inhibition of neutrophil chemotaxis, phagocytosis, and intracellular killing of pathogens. 26 Overall, diabetes and corticosteroid usage in COVID-19 patients leads to a high risk of invasive fungal infections.

An early diagnosis of mucormycosis is of utmost importance to improve outcomes in terms of survival, extent of surgical resection, disfigurement, and suffering. 9 10 Identification of the risk factors, clinical assessment, early use of imaging modalities, and quick initiation of diagnostic tests is essential for the early diagnosis of this pathology. Laboratory diagnosis of mucormycosis includes histopathology, direct examination of wet mounts and culture. Histopathology of the biopsy specimen from the affected tissue provides the definitive diagnosis of mucormycosis. Histopathological features include nonseptate or minimally septate, broad, ribbon-like hyphae (10 to 20 micrometers) invading the blood vessels. 11 Tissue biopsy specimens should also be sent for culture for a complete diagnostic workup, but the growth of this fungus is so slow in culture that the patient may expire long before the results are obtained.

On histopathological evaluation, the differentiation of mucormycosis from other fungi like aspergillus can also be made. In the present study, 76.3% cases were reported as mucormycosis on H&E-stained tissue sections with the help of special stains. Angioinvasion was present in 15.8% of the cases. However, 23.7% of the cases were reported as negative for fungal infestation on the tissue section even after multiple serial sectioning of the tissue. The fungal culture of these cases was positive.

There are a few limitations to our study. The study included a limited number of cases from a single center and may not represent the full picture of the disease burden of the world. We tried to identify the co-relation of diabetes and COVID-19 as a risk factor of mucormycosis and did not have sufficient data for other risk factors like AIDS/HIV, malignancy, neutropenia, or organ transplant. We did not compare our findings with a control group of patients who had mucormycosis but did not suffer from COVID-19 infection. We also do not have a long follow-up data. However, we have provided useful insights on the demographic and clinical profile of mucormycosis in COVID-19 patients and its relationship with predisposing factors.

## Conclusion

Mucormycosis is an angioinvasive fungal disease with significant morbidity and mortality. Recently, there has been a dramatic surge of the disease burden due to the interplay of COVID-19 infection, uncontrolled diabetes, and inappropriate corticosteroid usage. Histopathological evaluation is the cornerstone of diagnosis. Presence of broad, irregular, aseptate and right-angle branching hyphae on H&E-stained tissue sections, points towards the diagnosis of mucormycosis. Early detection, prompt surgical intervention, and antifungal medications lead to better survival rates.
